# Enhanced Synthesis of Polyphenols and Terpenes by UV-A Irradiation in *Artemisia argyi* Leaves

**DOI:** 10.3390/metabo16060367

**Published:** 2026-05-28

**Authors:** Shaozheng Li, Zikun Zhang, Lanqi Yang, Heyang Wang, Haike Gu, Junfeng Liu

**Affiliations:** 1 College of Life Science and Technology, Beijing University of Chemical Technology, Beijing 100029, China; 2 National Natural History Museum of China, Beijing 100050, China; 3 Institute of Radiation Technology, Beijing Academy of Science and Technology, Beijing 100875, China

**Keywords:** *Artemisia argyi*, secondary metabolite, UV-A radiation, phytohormone

## Abstract

**Background:** Secondary metabolites not only constitute the material basis for plant responses to multiple environmental stresses but are also extensively utilized in the pharmaceutical industry. **Methods:** In the present work, we investigated the metabolic response of *Artemisia argyi* to UV-A irradiation through transcriptomic and metabolomic analyses. **Results:** After 16 h of UV-A treatment with an intensity of 2.5 μmol m^−2^ s^−1^ and 8 h of dark cultivation, a total of 4343 differentially expressed genes were identified, most of which were associated with fatty acid metabolism, biosynthesis of secondary metabolites, and ribosome. Of the 1959 metabolites detected in samples exposed to a 16/8 h UV-A/dark cycle for 6 days, a total of 223 differentially accumulated metabolites were identified and classified into 12 subgroups, with phenolic acids and flavonoids representing the largest subgroups. Comprehensive analyses indicated that polyphenols and terpenes play critical roles in the adaptation of *A. argyi* to UV-A irradiation. The phytohormone methyl jasmonate was identified as a key regulator of the enhanced synthesis of these secondary metabolites, through activation of transcription factors from the MYB and bHLH families. **Conclusions:** This study deepens our understanding of secondary metabolic regulation in response to UV-A stress and provides a simple and reliable method to promote the accumulation of specific secondary metabolites in *Artemisia* species.

## 1. Introduction

*Artemisia argyi* H. Lév. & Vaniot is a perennial herb in the Asteraceae family, widely distributed in the temperate zone of the Northern Hemisphere. This plant is renowned for its extensive history of medicinal and culinary applications [1], as it is abundant in a variety of bioactive compounds, including essential oil, flavonoids, coumarins, organic acids, and polysaccharides [2,3]. These active constituents not only confer protection against biotic and abiotic stresses but are also widely used in the pharmaceutical, nutraceutical, and cosmetic industries [4,5,6]. Among these compounds, flavonoids and phenolic substances play a crucial role in regulating the production of reactive oxygen species (ROS), with their synthesis being significantly influenced by multiple abiotic stressors such as nutrient availability, pH levels, temperature fluctuations, and light conditions [7,8,9,10].

Solar light serves dual functions as both an energy source and a developmental signal for plants. Solar radiation encompasses ultraviolet (UV, 200–400 nm) light as well as photosynthetically active radiation (400–700 nm). Although only 7–9% of terrestrial sunlight falls within the UV spectrum, this portion exerts profound biological effects [11]. UV radiation is typically classified as UV-A (315–400 nm, fully reaching Earth’s surface), UV-B (280–315 nm, partially transmitted through ozone), and UV-C (100–280 nm, fully absorbed by the atmosphere) [11]. As the shortest wavelength that penetrates to the surface, UV-B acts both as an environmental stressor and developmental regulator [12,13]. Extensive research has been conducted on metabolic and morphological alterations in plants exposed to UV-B [7,14,15,16]. It is well documented that UV-B generally inhibits plant growth and development, whereas UV-A can promote the growth of *Arabidopsis thaliana*, implying distinct regulatory mechanisms between these two types of ultraviolet radiation [17,18]. UV-A modulates plant development primarily via cryptochrome 1 (cry1) signaling pathway, which regulates the expression of several genes involved in flavonoid biosynthesis and the circadian expression of chlorophyll a/b-binding protein genes [19]. Phytochrome B acts upstream of the convergence point where the UV-A/blue light synergistic pathways interact with the UV-B signaling pathway [20]. In addition, UV-A exposure has been shown to reduce photosynthetic efficiency in microalgae, while simultaneously elevating the accumulation of bioactive compounds in *Centella asiatica* leaves [21,22].

Nowadays, there is a growing interest in harnessing UV radiation to enhance the secondary metabolites of plants, including phenolic compounds, triterpenoids, alkaloids, and organic acids [23]. This strategy arises from protective and reparative mechanisms of plants in response to UV stress through endogenous physiological processes. High doses of UV radiation can cause DNA damage, protein denaturation, membrane peroxidation, and ROS accumulation, ultimately leading to oxidative stress [24]. In our recent studies, we found that UV-B radiation effectively enhances the synthesis of flavonoids and volatile compounds in *A. argyi* [7,25]. However, the effects of UV-A radiation on the metabolism of *A. argyi* remain unclear.

In this study, we conducted a multi-omics analysis to evaluate the impact of UV-A irradiation on gene expression and metabolites in *A. argyi*. Our findings provide deeper insights into how UV-A radiation influences the biosynthesis of secondary metabolites and offer a straightforward method for enhancing the economic value of this herbal plant.

## 2. Materials and Methods

### 2.1. Plant Material and Cultivation Method

The *A. argyi* cultivar utilized in this work was kindly gifted by Guoyizhongjing Wormwood Industry Group Co., Ltd. (Nanyang, China) and then propagated in our laboratory. These plants were cultivated in sandy loam in flower pots (30 cm diameter × 25 cm height) and grown in a growth chamber at 25 °C for two months under a 16 h light/8 h dark photoperiod, with a light intensity of 100 μmol m^−2^ s^−1^. No additional dark-adaptation period was implemented prior to UV-A treatment; plants were directly transferred from visible light (100 µmol m^−2^ s^−1^) to UV-A radiation (2.5 µmol m^−2^ s^−1^). Thereafter, leaf samples were taken at 0 h, 24 h, and 6 days. After rapid freezing with liquid nitrogen, the harvested leaf samples were stored at −80 °C for further analysis.

### 2.2. RNA Isolation and Sequencing

Total RNA was extracted from 6 samples collected at 0 h and 24 h using TRIzol reagent (Invitrogen, Carlsbad, CA, USA) following the manufacturer’s protocol. RNA purity and quantification were determined with a NanoDrop 2000 spectrophotometer (Thermo Scientific, Waltham, MA, USA), while RNA integrity was evaluated using an Agilent 2100 Bioanalyzer (Agilent Technologies, Santa Clara, CA, USA). RNA-seq libraries were subsequently constructed using VAHTS Universal V6 RNA-seq Library Prep Kit according to the manufacturer’s instructions. Transcriptome sequencing and data analysis were performed by OE Biotech Co., Ltd. (Shanghai, China).

The libraries were sequenced on a Novaseq 6000 platform (Illumina, San Diego, CA, USA) and 150 bp paired-end reads were generated. Using the *A*. *argyi* genome as a reference (https://ngdc.cncb.ac.cn/gwh/Assembly/30841/show, accessed on 2 November 2023), raw reads of fastq format were firstly processed using fastp and the low-quality reads were removed to obtain the clean reads [26]. FPKM of each gene was calculated and the read counts of each gene were obtained by HTSeq-count [27]. Principal component analysis (PCA) was performed using R (v 3.2.0) to evaluate the biological duplication of samples. Differential expression analysis was performed employing the DESeq2 (v 1.40.5). DEGs between different groups were screened with thresholds of |log_2_(fold change)| ≥ 1 and q-value < 0.05. Based on the hypergeometric distribution, GO, KEGG pathway, Reactome and WikiPathways enrichment analysis of DEGs were performed to screen the significant enriched terms using R (v 3.2.0). Samples taken at 0 h were used as the control.

### 2.3. Metabolite Extraction and Widely Targeted Metabolome Analysis

A total of 6 samples harvested at 0 h and 144 h were freeze-dried using a Scientz-100F lyophilizer (Scientz Biotechnology Corporation, Ningbo, China). The dried tissues were finely ground with a mixer mill (MM400, Retsch, Haan, Germany) using a zirconia bead at 30 Hz for 1.5 min. Subsequently, 50 mg of the resulting powder was extracted with 1.2 mL of precooled 70% (*v*/*v*) methanol. The mixture was vortexed for 30 s at 30 min intervals, repeated six times in total to ensure thorough extraction. After centrifugation at 12,000 *g* for 3 min, the supernatants were collected and passed through a 0.22 μm SCAA-104 membrane filter (ANPEL, Shanghai, China) prior to widely targeted metabolomic analysis (WTMA, Waynesboro, PA, USA).

The sample extracts were analyzed using a Triple Quad 6500+ UPLC-ESI-MS/MS system (SCIEX, Washington, DC, USA), as previously reported [28]. WTMA was performed by Metware Biotechnology Corporation (Wuhan, China). Quality control (QC) samples were injected every three experimental samples (3 QC samples in total) to monitor instrument stability. Both positive and negative electrospray ionization modes were employed. Unsupervised PCA was performed by statistics function prcomp within R (www.r-project.org, accessed on 6 November 2023). The data was unit variance scaled before unsupervised PCA. For two-group comparisons, differential accumulated metabolites (DAMs) were identified based on variable importance in projection (VIP > 1) derived from orthogonal partial least squares-discriminant analysis (OPLS-DA) and absolute Log_2_ Fold Change values (|log_2_FC| ≥ 1). The identified metabolites were annotated using KEGG Compound database (http://www.kegg.jp/kegg/compound, accessed on 7 November 2023), annotated metabolites were then mapped to KEGG Pathway database (http://www.kegg.jp/kegg/pathway.html, accessed on 6 November 2023). 2-chlorophenylalanine was used as internal standard. Three biological replicates were employed in each group.

### 2.4. Quantitative Real-Time PCR

To validate the reliability of the transcriptomic data, quantitative real-time PCR (qRT-PCR) was conducted on a selected set of genes. Total RNA was extracted using the Plant RNA Kit (Omega, New York, NY, USA) and subsequently reverse-transcribed into cDNA with the UEIris II RT-PCR Kit (Novoprotein, Suzhou, China). The resulting cDNA served as the template for qRT-PCR analysis, which was performed using the NovoStart SYBR qPCR SuperMix Plus system (Novoprotein, China). Gene-specific primers used in the qRT-PCR assays are listed in Table S1. All experimental procedures were carried out in strict accordance with the manufacturers’ protocols. Relative gene expression levels were calculated using the 2^−ΔΔ*C*T^ method [29].

### 2.5. Statistical Analysis

All treatments were performed with three biological replicates, and the results are presented as mean ± standard deviation (SD). Statistical significance was determined using a Student’s *t*-test, with differences considered significant at *p* < 0.05.

## 3. Results and Discussion

### 3.1. RNA Sequencing and DEG Analysis

**Figure 1:**
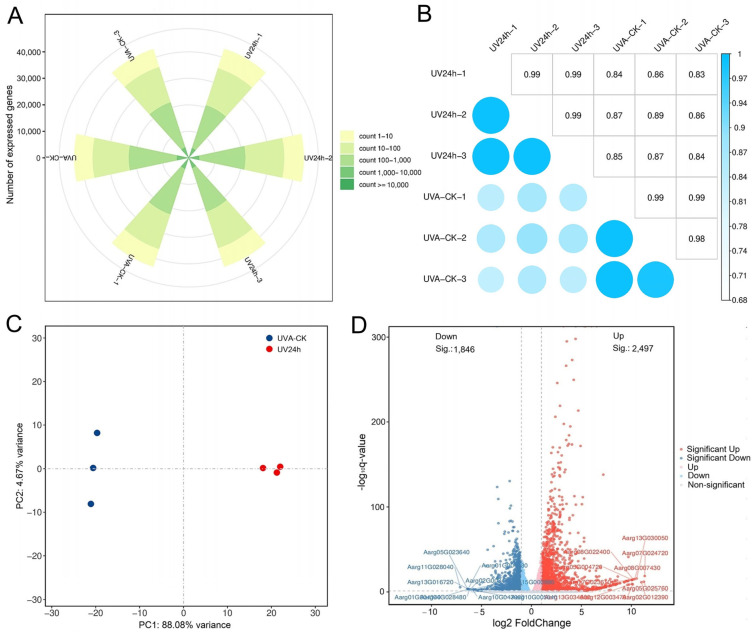
Overview of transcriptome analysis of *A. argyi* responsive to UV-A irradiation. (**A**) Gene number in each sample. (**B**) Heat map of correlation coefficient between samples. The horizontal and vertical axes represent the sample name, and the color represents the size of the correlation coefficient. (**C**) PCA analysis of samples taken at 0 (CK) and 24 h. (**D**) Volcano map of DEGs.

To elucidate the potential molecular mechanism underlying *A. argyi*’s response to UV irradiation, RNA-Seq analysis was carried out on samples subjected to UV-A irradiation for 0 h (control) and 24 h. The 24 h transcriptome, obtained under a 16 h UV-A/8 h dark photoperiod, was representative of transcriptional changes in *A. argyi* genes and could also predict 6-day metabolomic results, though with some limitations. The mapping ratio of sample reads to the reference genome, along with the quality preprocessing outcomes of the sequencing data, demonstrated that the sequencing data were suitable for further analysis (Tables S2 and S3). By screening genes encoding proteins, approximately 43,200 genes were identified in each sample (Figure 1A, Table S4). Correlation analysis among samples revealed a high correlation within groups (0.99), while correlations between different groups were relatively lower (0.83–0.89), thereby confirming the efficacy of UV-A radiation treatment (Figure 1B). Principal component analysis (PCA) demonstrated that PC1 accounted for 88.08% of the variation and effectively separated UV-treated samples from controls, whereas PC2 explained only 4.67% of the variance and distinguished changes among samples within each group (Figure 1C). Differentially expressed genes (DEGs) were identified based on the value of log2(Fold change) and *p*-value. Specifically, there were 2497 upregulated DEGs and 1846 downregulated DEGs observed in total (Figure 1D). Notably, the number of upregulated DEGs following UV-A treatment exceeded that observed with UV-B treatment, while the count of downregulated DEGs remained comparable [7]. This disparity in upregulated DEGs reflected distinct physiological and metabolic responses of *A. argyi* to exposure to UV-A versus UV-B radiation [2,30]. Furthermore, these DEGs might play a crucial role in mediating *A. argyi*’s response to UV-A irradiation.

### 3.2. Metabolic Pathway Enrichment for DEGs

**Figure 2:**
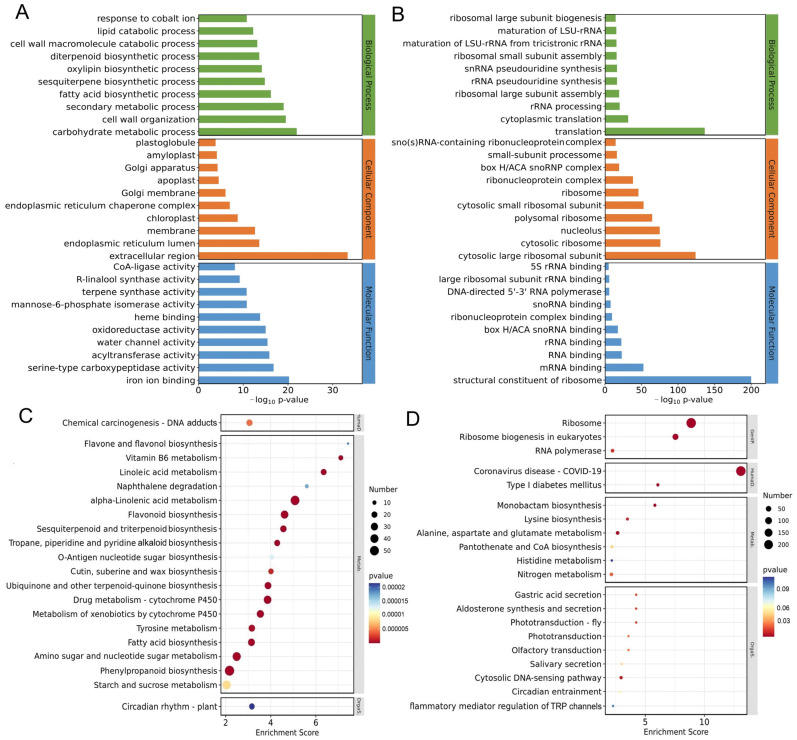
Enrichment analysis of DEGs. (**A**) Top 30 upregulated items obtained from GO enrichment. (**B**) Top 30 downregulated items obtained from GO enrichment. (**C**) Top 20 upregulated items obtained from KEGG enrichment. (**D**) Top 20 downregulated items obtained from KEGG enrichment.

The DEG-enriched biological processes were identified using the GO and KEGG databases. The GO enrichment analysis revealed that the most significantly upregulated “biological process” included “carbohydrate metabolic process”, “cell wall organization”, “secondary metabolic process”, “sesquiterpene biosynthetic process”, “fatty acid biosynthetic process”, etc. (Figure 2A). Overall, lipid and terpene metabolism emerged as the primary upregulated categories. The most significantly upregulated “cellular component” was predominantly associated with the “extracellular region”, “endoplasmic reticulum”, “membrane”, and “chloroplast”. In terms of “molecular function”, the items exhibiting the highest levels of upregulation included “iron ion binding”, “serine-type carboxypeptidase activity”, and “acyltransferase activity”. Conversely, the most significantly downregulated categories in terms of “biological process”, “cellular component”, and “molecular function” were identified as “translation”, “cytosolic ribosome”, and “structural constituent of ribosome”, respectively (Figure 2B). This suggested that UV-A radiation had a substantial negative impact on protein synthesis processes. Through KEGG enrichment analysis, lipid metabolism pathways such as “alpha-linolenic acid metabolism”, “linoleic acid metabolism”, and “fatty acid biosynthesis” were found to be among the most significantly enriched pathways (Figure 2C). These findings aligned with previous studies indicating that rapid alterations in membrane lipids, particularly unsaturated fatty acids, are essential for cells to adapt to diverse environmental stresses [31]. Following was “biosynthesis of other secondary metabolites”, which includes pathways such as “flavonoid biosynthesis”, “tropane, piperidine and pyridine alkaloid biosynthesis”, “phenylpropanoid biosynthesis”, and “flavone and flavonol biosynthesis”. Given that secondary metabolites like flavonoids and alkaloids are typically synthesized in response to various stressors [32,33,34], it is plausible that the upregulated DEGs involved in these pathways may play a critical role in *A. argyi*’s resistance to UV-A radiation. The enrichment of “Chemical carcinogenesis-DNA adducts” pathway indicated that UV-A irradiation imposed oxidative stress on *A. argyi*, potentially leading to ROS-mediated DNA damage. Consequently, DNA repair and protective metabolic pathways were activated to maintain genomic stability and cellular homeostasis. Interestingly, “circadian rhythm-plant” was also identified as an enriched item, indicating that UV-A interfered with the circadian rhythm of *A. argyi* [35]. The significantly downregulated terms “ribosome” and “ribosome biogenesis in eukaryotes” were consistent with the results obtained from GO analysis (Figure 2D). The downregulation of ribosome and its biosynthetic pathway could reduce the energy consumption of protein synthesis, thereby redirecting more carbon sources, ATP, and reducing equivalents toward the production of defense-related secondary metabolites. This “trade-off” metabolic reprograming enables plants to rapidly establish chemical defense barriers under stress conditions, representing an adaptive regulatory mechanism that sacrifices some growth potential for survival advantage [36,37]. In brief, transcriptome data suggested that the biosynthesis of secondary metabolites and fatty acid metabolism might play a crucial role in the adaptation of *A. argyi* to UV-A radiation.

### 3.3. Metabolomic Analysis

**Figure 3:**
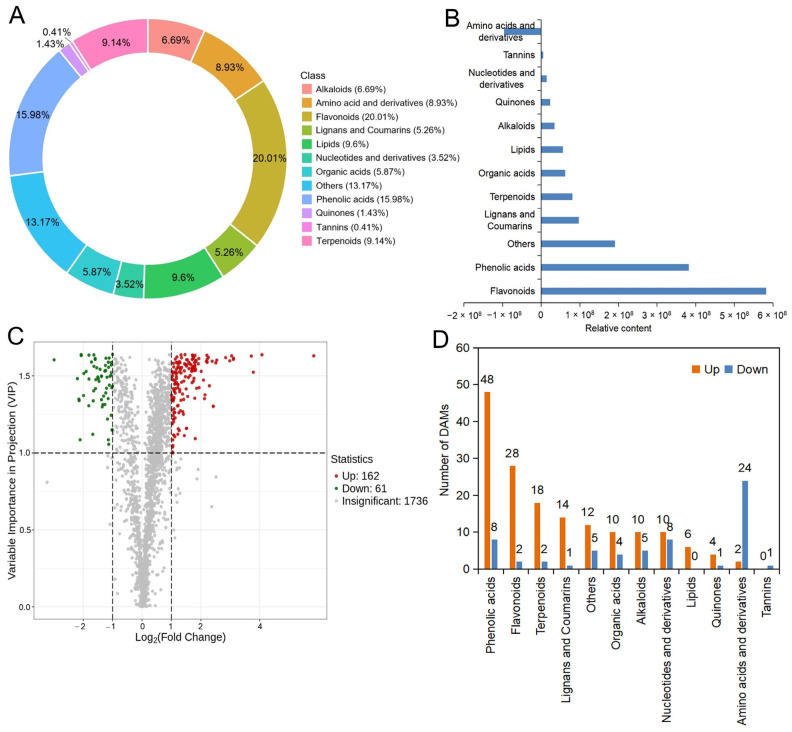
Overview of the metabolite changes in *A. argyi* leaves in response to UV-A radiation. (**A**) Category and percentage of metabolites. Twelve colored squares on the left represent different class of metabolites. (**B**) Relative content of metabolites. The relative content is defined as the total relative content of a specific subgroup of metabolites in UV6d, subtracted by the total relative content of the same subgroup of metabolites in UV0. (**C**) Volcano plot of metabolites. (**D**) Category and number of DAMs.

To validate the transcriptome findings, a widely targeted metabolomics analysis was conducted on samples collected at 0 h and 6 days following UV-A irradiation. PCA showed that PC1 accounted for 49.69% of the variation and effectively distinguished between UV-treated samples and controls; meanwhile, PC2 explained 20.91% of the variation by differentiating changes within each sample group (Figure S1). The detected metabolites are classified based on their chemical structure, biosynthetic pathway, and functional attribution. A total of 1959 metabolites were detected across all samples, which could be classified into 12 subgroups: flavonoids (20.01%), phenolic acids (15.98%), lipids (9.6%), terpenoids (9.14%), amino acids and derivatives (8.93%), alkaloids (6.69%), organic acids (5.87%), lignans and coumarins (5.26%), nucleotides and derivatives (3.52%), quinones (1.43%), and tannins (0.41%), among others (13.17%) (Figure 3A, Table S5). Among these subgroups, flavonoids and phenolic acids were the two categories with the highest relative content upregulation in UV-exposed mugwort leaves, while amino acids and derivatives are the only category with a relative content decrease (Figure 3B). Employing a threshold of |log2Fold Change| ≥ 1 and VIP > 1, a total of 223 differentially accumulated metabolites (DAMs) were identified in the comparison between UV-A6d and control, comprising 162 upregulated DAMs and 61 downregulated DAMs (Figure 3C).

Further analysis revealed that compounds exhibiting the highest number of upregulation included phenolic acids (40), flavonoids (26), and terpenes (16), as well as lignans and coumarins (13) (Figure 3D). As is well known, these significantly upregulated secondary metabolites play critical signaling and structural molecular roles in plant defense and development [7,14]. It was noteworthy that the highest net reduction in DAMs was observed in “amino acids and derivatives”. This phenomenon was likely attributed to the downregulation of “translation” and the enhanced synthesis of secondary metabolites utilizing amino acids as feedstock. Collectively, secondary metabolites, including phenolic acids, flavonoids, and terpenes, were inferred to play a critical role in the response of *A. argyi* to UV-A radiation.

### 3.4. Candidate DAMs with Potential Roles in UV-A Resistance

**Figure 4:**
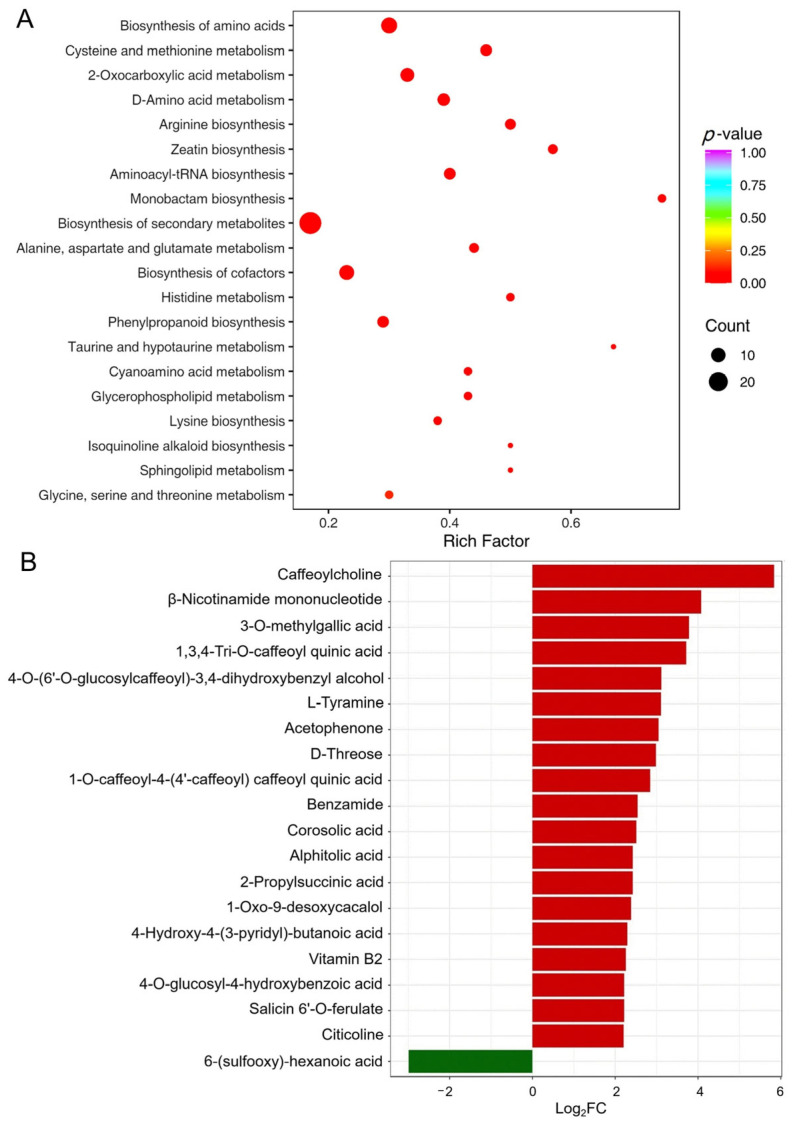
Enrichment analysis of DAMs. (**A**) Enrichment pathways of DAMs. (**B**) Top 20 DAMs.

Amino acids function not only as fundamental building blocks for protein synthesis but also as key precursors in the biosynthesis of diverse secondary metabolites, including flavonoids, phenylpropanoids, alkaloids, and phenolic acids. KEGG enrichment analysis revealed that the significantly enriched pathway “biosynthesis of amino acids” underpins the pronounced upregulation observed in both “biosynthesis of secondary metabolites” and “phenylpropanoid biosynthesis” (Figure 4A). Cofactors serve as essential participants in numerous enzyme-catalyzed reactions and are indispensable for maintaining enzymatic activity. The significant enrichment of the “biosynthesis of cofactors” pathway further corroborates the activation of “biosynthesis of amino acids” and “biosynthesis of secondary metabolites”. Furthermore, the significantly enriched pathway “2-oxocarboxylic acid metabolism” might enhance plant adaptability to UV radiation through synergistic interactions with secondary metabolites [38]. Among the 20 DAMs exhibiting the largest fold changes, 19 were upregulated and only one was downregulated (Figure 4B). The upregulated DAMs comprised six phenolic acids (3-O-methylgallic acid, 4-O-(6′-O-glucosylcaffeoyl)-3,4-dihydroxybenzyl alcohol, 1,3,4-tri-O-caffeoyl quinic acid, 1-O-caffeoyl-4-(4′-caffeoyl) caffeoyl quinic acid, 4-O-glucosyl-4-hydroxybenzoic acid, and salicin 6′-O-ferulate), four alkaloids (caffeoylcholine, 4-hydroxy-4-(3-pyridyl)-butanoic acid, L-tyramine, and benzamide), three terpenoids (alphitolic acid, corosolic acid, and 1-oxo-9-desoxycacalol), one nucleotide (β-nicotinamide mononucleotide), and one organic acid (2-propylsuccinic acid), along with four others (acetophenone, D-threose, vitamin B2, and 6-(sulfooxy) hexanoic acid). Surjadinata et al. (2017) report that UV-A, UV-B, and UV-C radiation can affect the types of phenolic compounds produced in fresh-cut carrots, among which UV-A does not induce the generation of *p*-coumaric acid derivative and *p*-hydroxybenzoic acid derivative [39]. However, *p*-coumaric acid derivative and *p*-hydroxybenzoic acid derivative were identified as the most significantly upregulated DAMs in the current study, highlighting notable metabolic differences among various plant species. Additionally, secondary metabolites such as phenolic acids, alkaloids, terpenoids, and nucleotide derivatives possess the ability to absorb UV radiation and scavenge free radicals [40,41,42], thereby enhancing the plant’s resilience against environmental stressors.

### 3.5. Analysis of Secondary Metabolite Biosynthesis

**Figure 5:**
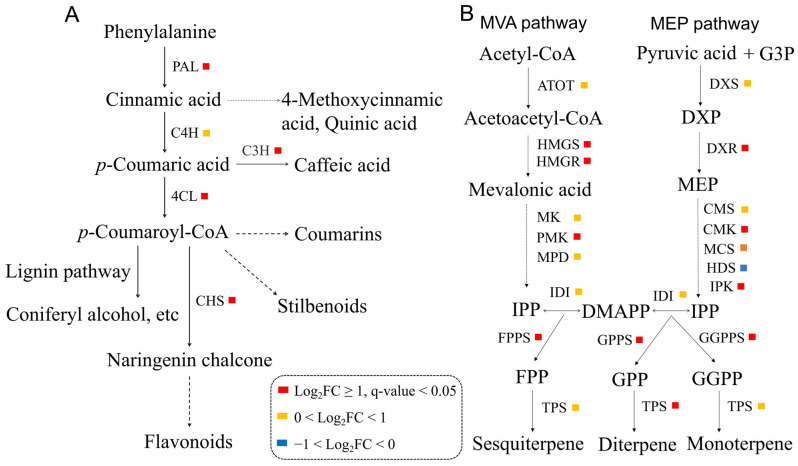
Expression of genes involved in the synthesis of polyphenols (**A**) and terpenes (**B**) in *A. argyi* leaves exposed to UV radiation. PAL, phenylalanine ammonia-lyase; C4H, cinnamate 4-hydroxylase; 4CL, 4-coumarate-CoA ligase; CHS, chalcone synthase; C3H, p-coumarin-3-hydroxylase; ATOT, acetoacetyl-CoA nliolase; HMGS, hydroxymethylglutaryl-CoA synthase; HMGR, hydroxymethylglutaryl-CoA reductase; MK, mevalonate kinase; PMK, phosphomevalonate kinase; MPD, mevalonate pyrophosphate decarboxylase; G3P, glyceraldehyde 3-phosphate; DXS, deoxyxylulose 5-phosphate synthase; DXP, 1-deoxy-2-xylulose-5-phosphate; DXR, deoxyxylulose phosphate reductase; MEP, 2-C-methyl-D-erythritol-4-phosphate; CMS, 4-diphosphocytidyl-2-C-methyl-D-erythritol synthase; CMK, 4-diphosphocytidyl-2-C-methyl-D-erythritol kinase; MCS, 2-C-methyl-D-erythritol 2,4-cyclodiphosphate synthase; HDS, 1-hydroxy-2-methyl-2-(E)-butenyl 4-diphosphate synthase; IPK, isopentenyl monophosphate kinase; IDI, isopentenyl diphosphate isomerase; IPP, isopentenyl pyrophosphate; DMAPP, dimethylallyl pyrophosphate; FPP, farnesyl pyrophosphate; GPP, geranyl diphosphate; GGPP, geranylgeranyl diphosphate; FPPS, farnesyl diphosphate synthase; GPPS, geranyl diphosphate synthase; GGPPS, geranylgeranyl diphosphate synthase; TPS, terpene synthase.

Environmental factors like UV-B radiation and physical damage have been shown to elevate phenolic acid levels in plants [43], underscoring their critical role in plant stress responses. Phenolic acids are primarily synthesized through the phenylpropanoid pathway in plants, which initiates with phenylalanine and tyrosine derived from the shikimate pathway. Concurrently, other phenylpropanoid compounds, including lignin, flavonoids, anthocyanins, and tannins, are also produced via this pathway [44]. Genes encoding key enzymes such as phenylalanine ammonia-lyase (PAL), 4-coumarate-CoA ligase (4CL), cinnamate4-hydroxylase (C4H), and p-coumarin-3-hydroxylase (C3H) exhibited significant upregulation or increased expression (Figure 5A). This confirmed a marked increase in phenolic acids (including 4-methoxycinnamic acid, caffeic acid, etc.), phenolic compounds (such as coumarins and stilbenoids), as well as lignins like coniferyl alcohol under UV-A radiation exposure. Moreover, chalcone synthase (CHS), which is responsible for synthesizing chalcone, was significantly upregulated. This facilitated the production of various flavonoids from chalcone precursor. As the most extensively studied secondary metabolites in plant defense systems, flavonoids can reduce ROS generated by electron transport chain dissipation while directly reducing UV-induced oxidative damage [45,46]. The results obtained from qRT-PCR analyses for PAL, CHS, 4CL, and C3H corroborated findings from RNA-seq (Figure S2). The upregulation of these pivotal genes aligned with metabolomic analysis outcomes, indicating that phenolic acids, flavonoids, coumarins, and lignins were enhanced in *A. argyi* leaves subjected to UV-A radiation.

Terpenes represent the largest family of secondary metabolites found in plants, which can be classified into hemiterpenes, monoterpenes, homoterpenes, sesquiterpenes, diterpenes, sesterpenes, triterpenes, tetraterpenes and polyterpenes [47]. Although these compounds are not essential for plant growth and development, they play a crucial role in regulating the interactions between plants and their environment [47]. Terpene biosynthesis occurs via two primary pathways: the mevalonic acid (MVA) pathway and 2-C-methyl-D-erythritol-4-phosphate (MEP) pathway. Hydroxymethylglutaryl-CoA reductase (HMGR) serves as the critical rate-limiting enzyme in the MVA pathway, while deoxyxylulose 5-phosphate synthase (DXS) and deoxyxylulose phosphate reductase (DXR) function as rate-limiting enzymes within the MEP pathway. The upregulation or significant upregulation of these key enzymes indicated an enhancement in the upstream synthesis pathways of terpenes (Figure 5B). Meanwhile, there was a notable increase in the expression levels of farnesyl diphosphate synthase (FPPS), geranyl diphosphate synthase (GPPS), and geranylgeranyl diphosphate synthase (GGPPS), which catalyze the formation of intermediate products FPP, GPP, and GGPP, respectively, was significantly upregulated. Additionally, terpene synthase (TPS) responsible for synthesizing diterpenes was significantly upregulated, while TPSs responsible for synthesizing monoterpenes and sesquiterpenes exhibited increased expression levels. In terms of the total accumulation of different terpene classes, triterpenes exhibited the most pronounced increase, followed by monoterpenes and sesquiterpenes (Figure S3). This observation suggested that diterpenes may undergo further metabolic conversion to generate other terpenes and their derivatives. Another possible reason is that the expression of TPS responsible for synthesizing diterpenes is downregulated after 24 h of UV-A treatment.

### 3.6. The Regulation of Phytohormones

**Figure 6:**
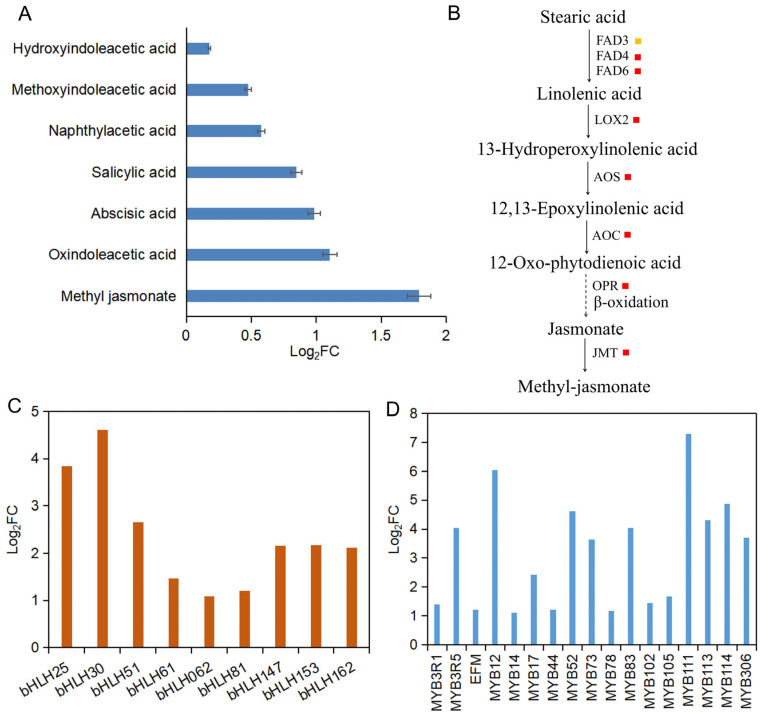
The role of phytohormones in *A. argyi* leaves exposed to UV-A radiation. (**A**) Changes in phytohormones in *A. argyi* leaves. (**B**) Relative expression levels of genes involved in methyl-JA biosynthesis. Relative expression levels of transcription factor bHLH (**C**) and MYB (**D**) families. FAD3, omega-3 fatty acid desaturase; FAD4, fatty acid desaturase 4; FAD6, omega-6 fatty acid desaturase; LOX2, lipoxygenase 2; AOS, allene oxide synthase; AOC, allene oxide cyclase; OPR, 12-oxophytodienoate reductase; JMT, jasmonic acid carboxyl methyltransferase.

Plant hormones are vital regulators of plant growth, development, and physiological processes. Amongst the phytohormones analyzed in this study, only methyl jasmonate showed significant upregulation (Figure 6A). Abscisic acid, salicylic acid, and naphthylacetic acid were observed to be moderately elevated as well. Notably, exposure to UV-A resulted in increased or significantly elevated levels of oxindoleacetic acid, methoxyindoleacetic acid, and hydroxyindoleacetic acid, indicating extensive conversion of indoleacetic acid into its inactive form. Changes in plant hormones suggested that methyl jasmonate synergistically regulated the metabolic response of *A. argyi* to UV-A irradiation, in conjunction with salicylic acid, abscisic acid, naphthylacetic acid, and indoleacetic acid. Previous studies have shown that methyl jasmonate enhances the production of phenolic acids and flavonoids in plants [7,48,49], thereby regulating the synthesis of secondary metabolites in *A. argyi*. Transcriptome analysis revealed that all genes involved in the synthesis of methyl jasmonate from stearic acid were significantly upregulated, with the exception of FAD3, which was not significantly upregulated (Figure 6B). This provided a plausible explanation for the observed increase in methyl jasmonate levels.

The MYB, bHLH, and WD40 families function as key regulators for synthesizing secondary metabolites such as flavonoids, phenolic acids, terpenoids, and alkaloids in plants [50,51,52,53]. Among these three transcription factor families, 9 bHLH familiy members, including bHLH30, bHLH25, bHLH51, bHLH147, etc., as well as 17 MYB familiy members, such as MYB111, MYB12, MYB52, and MYB114, were found to be significantly upregulated (Figure 6C,D). This finding was further confirmed by qRT-PCR (Figure S4). In comparison to results obtained from UV-B-exposed leaves of *A. argyi* [7,25], a greater number of members from both the bHLH and MYB families exhibited significant upregulation under UV-A irradiation, which might contribute to the synthesis of more metabolites, including flavonoids, phenylpropanoids/lignin, terpenes, alkaloids, glucosinolates, and phenolic acids. In summary, these findings suggest that methyl jasmonate regulates the expression of PAL, 4CL, CHS, HMGR, and DXR, among other genes, by activating transcription factors belonging to the MYB and bHLH families, thereby enhancing the synthesis of secondary metabolites and aiding *A. argyi*’s adaptation to UV-A exposure.

## 4. Conclusions

In this study, metabolites and genes encoding key enzymes and regulators in *A. argyi* exposed to UV-A irradiation were identified through transcriptomic and metabolomic analyses. A total of 4343 DEGs and 223 DAMs were detected. The DEGs were predominantly enriched in “lipid metabolism”, “biosynthesis of secondary metabolites” and “ribosome”. Most DAMs consisted of secondary metabolites, including phenolic acids, flavonoids, terpenes, lignins and coumarins. Through comprehensive analysis, the synthesis of secondary metabolites was primarily regulated by methyl jasmonate, which activated MYB and bHLH family members to regulate the expression of key enzymes such as PAL, 4CL, CHS, HMGR, DXR, etc. This work provides valuable insights into the metabolic changes induced by UV-A irradiation in plants while offering an accessible strategy for enhancing specific secondary metabolites in medicinal herbs.

## Data Availability

The data presented in this study are available on request from the corresponding author.

## Supplementary Materials

The following supporting information can be downloaded at: https://www.mdpi.com/article/10.3390/metabo16060367/s1, Figure S1: PCA of all samples; Figure S2: Validation of expression of key enzymes by qRT-PCR. Nine enzymes were selected: PAL, CHS, 4CL, C3H, DXR, HMGR, JMT, TPS, LOX2. Actin was used as control.; Figure S3: Changes in terpene content after UV-A treatment. Figure S4: Validation of expression of key transcription factors by qRT-PCR. Six transcription factors were selected: bHLH25, bHLH30, bHLH81, MYB12, MYB111, and MYB78. Actin was used as control.; Figure S5: A workflow diagram of this study; Table S1: Primers for qRT-PCR analysis; Table S2: Comparison rate between sample reads and reference genome; Table S3: Preprocessing results of sequencing data quality; Table S4: Gene number in the sample; Table S5: Secondary metabolites identified in *A. argyi* leaves.
